# Prevalence and clinical correlates of diabetes in hospitalized heart failure patients: a retrospective study

**DOI:** 10.3389/fendo.2026.1806046

**Published:** 2026-04-23

**Authors:** Lingqin Li, Xinzhu Yuan, Yanni Zhang, Quanbo Zhang, Yufeng Qing

**Affiliations:** 1 Department of Rheumatology and Immunology, Affiliated Hospital of North Sichuan Medical College, Nanchong, China; 2 Department of Nephrology, Beijing Anzhen Nanchong Hospital of Capital Medical University, Nanchong Central Hospital, Nanchong, China; 3 Department of Nephrology, Affiliated Hospital of North Sichuan Medical College, Nanchong, China; 4 Department of Geriatrics, Affiliated Hospital of North Sichuan Medical College, Nanchong, China

**Keywords:** clinical correlates, diabetes, heart failure, machine learning, prevalence

## Abstract

**Background:**

Heart failure (HF) and diabetes frequently coexist and are associated with worse outcomes, yet contemporary evidence on the prevalence and clinical correlates of diabetes in hospitalized patients with HF in China remains limited.

**Methods:**

Data for this study were obtained from Zigong Fourth People’s Hospital. A total of 2,008 adults hospitalized with HF were included, of whom 466 had diabetes. Demographic, clinical, hemodynamic and laboratory variables were extracted from the first admission records. We estimated the prevalence of diabetes and used univariate and multivariable logistic regression to identify factors associated with diabetes. Machine learning models were applied to rank variable importance, and restricted cubic splines were used to explore the shapes of these associations. Sex- and age-stratified analyses were further conducted to assess heterogeneity.

**Results:**

The prevalence of diabetes among hospitalized patients with HF was 23.21% (95% CI: 21.39–25.13). In multivariable models, female sex, higher systolic blood pressure, statin use, elevated white blood cell (WBC) count, and higher serum potassium were associated with greater odds of diabetes, whereas higher high-density lipoprotein cholesterol (HDL-C) was inversely associated with diabetes (OR = 0.53, 95% CI: 0.37–0.76). WBC, potassium and HDL-C consistently ranked among the top contributors in both machine learning models. Restricted cubic splines revealed a nonlinear association between WBC count and diabetes, while potassium and HDL-C showed monotonic relationships. In stratified analyses, potassium and HDL-C remained associated with diabetes in females and in patients aged ≥70 years.

**Conclusion:**

In Chinese inpatients with HF, approximately one in four had concomitant diabetes. WBC count, blood pressure, potassium and HDL-C were associated with diabetes status, with some variation across sex and age groups. These findings provide additional evidence on the clinical characteristics associated with diabetes in hospitalized patients with HF.

## 1 Introduction

Heart failure (HF) and diabetes are two highly prevalent chronic conditions that frequently coexist and jointly worsen prognosis (1, 2). Globally, up to one quarter of patients with HF have concomitant diabetes, and in many contemporary cohorts the proportion reaches 20%–40%, reflecting the shared background of aging, obesity and cardiometabolic risk (3). When HF and diabetes occur together, patients experience higher rates of hospitalization, arrhythmia, renal dysfunction and premature death than those with either condition alone, and treatment becomes more complex due to polypharmacy and drug-disease interactions (4, 5). Previous studies have shown that diabetes in patients with HF is associated with a more complex clinical profile and worse health status, underscoring the importance of better characterizing this subgroup in routine clinical practice (6).

However, several important knowledge gaps remain. Despite the high prevalence and substantial clinical impact of diabetes in patients with HF, evidence specifically describing the prevalence of diabetes and its associated clinical characteristics among hospitalized patients with HF in real-world settings remains limited (7). In particular, data from Chinese inpatient populations are still relatively scarce, and, due to differences in demographic characteristics, comorbidity patterns, and healthcare practices, findings from Western cohorts may not be directly generalizable to Chinese patients (8). In addition, multiple studies have shown that clinical characteristics and routinely measured biomarkers such as sex, obesity, elevated blood pressure, renal impairment, pro-inflammatory markers and lipids are closely associated with diabetes in broader populations (9–11). However, whether these factors exhibit similar association patterns in patients with established HF, and which of them are most strongly related to diabetes in this specific clinical context, has not been sufficiently characterized in hospitalized patients with HF, particularly across clinically relevant subgroups such as sex and age.

Accordingly, this study was designed as a hospital-based cross-sectional analysis using an HF database derived from the electronic medical records of a tertiary care center in Southwest China to estimate the prevalence of diabetes and examine the clinical characteristics associated with diabetes in hospitalized patients with HF, with further analyses stratified by sex and age. By combining multivariable logistic regression, machine learning-based variable importance ranking, restricted cubic spline analyses, and stratified analyses, we further characterized the relative importance, association patterns, and subgroup heterogeneity of these factors.

## 2 Materials and methods

### 2.1 Data source

This study drew on a registry of consecutive HF hospitalizations at the Fourth People’s Hospital of Zigong spanning December 2016 to June 2019. The registry aggregates standardized variables extracted from the electronic medical record. A de-identified version was released publicly on PhysioNet by Zhang et al. (12, 13).

### 2.2 Study population

Patients were eligible if they were aged ≥18 years and had a clinical diagnosis of HF at hospital admission. HF was diagnosed by cardiologists according to the 2016 European Society of Cardiology guidelines, based on typical symptoms and signs, elevated natriuretic peptide levels, and objective evidence of cardiac structural or functional abnormalities on echocardiography. In the source database, diabetes status was available for all 2,008 patients. According to the source data documentation, diabetes was identified from prior medical history information documented in the admission notes at hospital admission. Therefore, all 2,008 HF inpatients were included in the present analysis, of whom 466 had diabetes and 1,542 did not.

### 2.3 Data collection

We collected demographic characteristics (age and sex), HF related information including heart failure type and New York Heart Association functional class (NYHA), and anthropometric data such as body mass index (BMI). Medication variables included statins, ACEI/ARB, diuretics, and beta-blockers. Vital signs included systolic blood pressure (SBP), diastolic blood pressure (DBP), and mean arterial pressure (MAP). Hematologic indices included white blood cell count (WBC), monocyte count (MONO), lymphocyte count, red blood cell count (RBC), hemoglobin, and platelet count. Biochemical and cardiac biomarkers included B-type natriuretic peptide (BNP), high-sensitivity cardiac troponin (hs-cTn), serum creatinine (SCr), serum uric acid (SUA), estimated glomerular filtration rate (eGFR), serum calcium, serum potassium, albumin, globulin, albumin-to-globulin ratio (A/G ratio), total bilirubin (TBIL), total cholesterol, low-density lipoprotein cholesterol (LDL-C), triglycerides, and high-density lipoprotein cholesterol (HDL-C). All laboratory measurements were obtained from the first routine blood tests performed after admission.

### 2.4 Statistical analysis

All statistical analyses were conducted using R software (version 4.3.3) and DecisionLinnc (version 1.0) (14). Continuous variables were expressed as mean ± standard deviation (SD), and categorical variables as counts and percentages. Baseline characteristics were compared between patients with and without diabetes using the t-test or Mann–Whitney U test for continuous variables, and the chi-square test for categorical variables, as appropriate. The overall prevalence of diabetes and its 95% confidence interval (CI), as well as subgroup-specific prevalences, were calculated, and differences between subgroups were evaluated using the chi-square test. Missing data were handled using multiple imputation.

To identify factors associated with diabetes, we used logistic regression models with diabetes status as the dependent variable. First, univariate logistic regression analyses were performed for each clinical and laboratory variable, and odds ratios (ORs) with 95% CI were calculated. Variables with a P value < 0.05 in univariate analyses or considered clinically relevant were then included in a multivariable logistic regression model to obtain adjusted ORs and 95% CIs. Stepwise selection was not performed. Clinical relevance was determined based on demographic characteristics, heart failure severity, and variables related to inflammation, renal function, electrolyte balance, nutritional status, lipid metabolism, and available medication use, as informed by prior clinical knowledge and previous literature. Variance inflation factors were used to assess multicollinearity among covariates, and highly collinear variables were not entered simultaneously into the same model. To assess the robustness of these associations, the multivariable analyses were further repeated in sex- and age-stratified subgroups.

To provide a complementary assessment of variable importance, we constructed machine learning models using extreme gradient boosting (XGBoost) and random forest based on the significant variables identified in the multivariable analysis. The dataset was randomly split into a training set and a validation set at a ratio of 7:3. Hyperparameters were optimized using grid search combined with five-fold cross-validation, with the area under the receiver operating characteristic curve (AUC) used as the evaluation metric. Shapley Additive Explanations (SHAP) were then applied to quantify the relative contribution of each variable and derive an importance ranking. These machine learning analyses were performed primarily as complementary approaches for variable importance ranking and interpretability.

Subsequently, for continuous variables that remained significantly associated with diabetes in the multivariable model, we used restricted cubic spline (RCS) functions to further characterize the shape of the associations between these variables and diabetes. RCS models were fitted using four knots placed at the 5th, 35th, 65th, and 95th percentiles, with the second knot used as the reference value. A two-sided P value < 0.05 was considered statistically significant.

## 3 Results

### 3.1 Baseline characteristics of patients

A total of 2,008 patients with HF were included in this study. As shown in **Table 1**, baseline comparisons between patients with and without diabetes showed no significant differences in age distribution or HF type. In contrast, sex and NYHA class differed significantly between the two groups. The diabetes group had a higher proportion of females and a greater proportion of patients classified as NYHA class IV. Regarding clinical and laboratory parameters, patients with diabetes tended to have slightly higher BMI and SBP, along with higher WBC, monocyte and lymphocyte counts, and increased levels of SCr, potassium and triglycerides. On the other hand, hemoglobin, albumin, A/G ratio, TBIL and HDL-C were modestly lower in the diabetes group (all P < 0.05). Statin use was also more frequent in the diabetes group. For most of the remaining variables, including DBP, MAP, RBC count, platelet count, and LDL-C, no obvious differences were observed between the two groups.

**Table 1:** Baseline characteristics of study population.

Variable	Non-diabetes (n=1542)	Diabetes (n=466)	P-value
Age, n (%)			0.925
<70	418 (27.11)	128 (27.47)	
≥70	1124 (72.89)	338 (72.53)	
Sex, n (%)			0.036
female	873 (56.61)	290 (62.23)	
male	669 (43.39)	176 (37.77)	
Type, n (%)			0.251
left	368 (23.87)	109 (23.39)	
right	44 (2.85)	7 (1.50)	
both	1130 (73.28)	350 (75.11)	
NYHA, n (%)			0.022
II	279 (18.09)	74 (15.88)	
III	814 (52.79)	225 (48.28)	
IV	449 (29.12)	167 (35.84)	
BMI (kg/m²)	21.18 ± 3.74	21.68 ± 4.00	0.013
SBP (mmHg)	130.40 ± 24.64	133.219 ± 25.00	0.031
DBP (mmHg)	76.61 ± 14.28	76.46 ± 15.06	0.852
MAP (mmHg)	94.540 ± 16.20	95.38 ± 16.72	0.329
WBC (×10^9^/L)	7.06 ± 3.33	8.13 ± 3.82	<0.001
MONO (×10^9^/L)	0.47 ± 0.23	0.50 ± 0.28	0.004
RBC (×10^12^/L)	3.87 ± 0.76	3.83 ± 0.78	0.336
Lymphocyte (×10^9^/L)	1.01 ± 0.58	1.11 ± 0.67	0.002
Hemoglobin (g/L)	115.69 ± 24.480	113.02 ± 24.62	0.041
Platelet (×10^9^/L)	144.49 ± 65.80	147.33 ± 61.96	0.411
BNP (pg/mL)	1285.50 ± 1365.81	1263.84 ± 1316.55	0.764
hs-cTn (pg/mL)	0.27 ± 1.97	0.34 ± 1.77	0.498
SCr (μmol/L)	105.87 ± 74.24	118.95 ± 92.81	0.002
SUA (μmol/L)	483.46 ± 171.04	481.56 ± 165.19	0.833
eGFR (mL/min/1.73 m²)	69.37 ± 35.18	66.35 ± 40.89	0.123
Calcium (mmol/L)	2.29 ± 0.18	2.30 ± 0.19	0.602
Potassium (mmol/L)	3.95 ± 0.68	4.09 ± 0.78	<0.001
Albumin (g/L)	36.67 ± 4.91	36.07 ± 5.18	0.027
Globulin (g/L)	28.48 ± 6.15	28.88 ± 5.74	0.220
A/G ratio	1.34 ± 0.31	1.29 ± 0.29	0.005
TBIL (μmol/L)	23.35 ± 17.63	20.69 ± 16.84	0.005
Cholesterol (mmol/L)	3.7 ± 1.06	3.70 ± 1.18	0.638
LDL-C (mmol/L)	1.86 ± 0.74	1.85 ± 0.81	0.848
HDL-C (mmol/L)	1.12 ± 0.35	1.05 ± 0.35	<0.001
Triglyceride (mmol/L)	1.09 ± 0.94	1.39 ± 1.43	<0.001
Statins, n (%)			<0.001
NO	956 (62.04%)	229 (49.14%)	
YES	585 (37.96%)	237 (50.86%)	
ACEI/ARB, n (%)			0.510
NO	944 (61.26%)	294 (63.09%)	
YES	597 (38.74%)	172 (36.91%)	
Diuretics, n (%)			0.856
NO	25 (1.62%)	7 (1.50%)	
YES	1516 (98.38%)	459 (98.50%)	
Beta-blockers, n (%)			0.943
NO	954 (61.91%)	290 (62.23%)	
YES	587 (38.09%)	176 (37.77%)	

NYHA, New York Heart Association; BMI, body mass index; SBP, systolic blood pressure; DBP, diastolic blood pressure; MAP, mean arterial pressure; WBC, white blood cell; MONO, monocyte; RBC, red blood cell; BNP, B-type natriuretic peptide; hs-cTn, high-sensitivity cardiac troponin; SCr, serum creatinine; SUA, serum uric acid; eGFR, estimated glomerular filtration rate; A/G ratio, albumin-to-globulin ratio; TBIL, total bilirubin; LDL-C, low-density lipoprotein cholesterol; HDL-C, high-density lipoprotein cholesterol; ACEI, Angiotensin-Converting Enzyme Inhibitor; ARB, Angiotensin II Receptor Blocker.

### 3.2 Prevalence of diabetes

**Figure 1:**
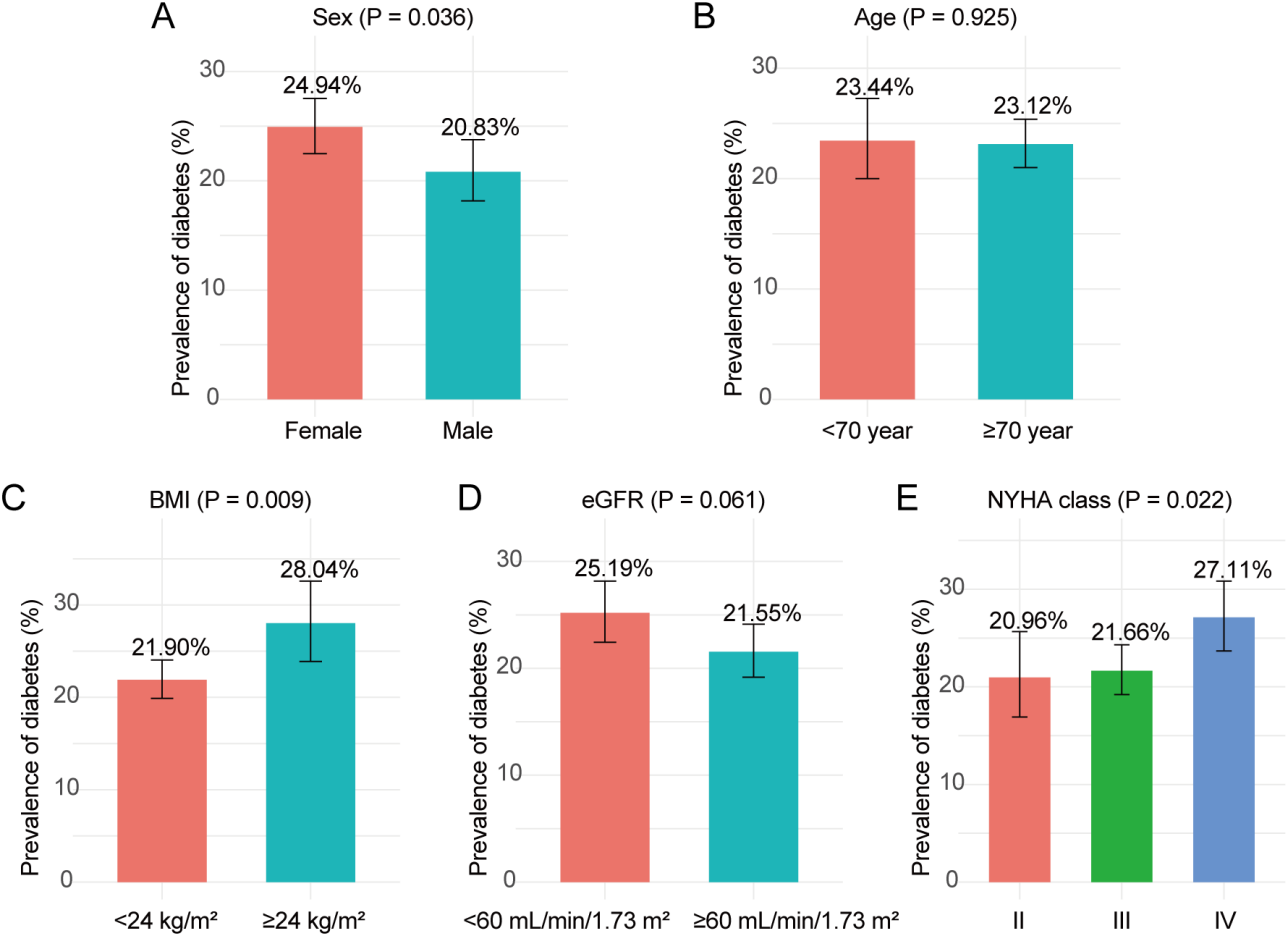
Prevalence of diabetes. Bar charts display the percentage of patients with Diabetes within each subgroup. **(A)** sex, **(B)** age, **(C)** BMI, **(D)** eGFR, and **(E)** NYHA. P-values refer to comparisons between subgroups. BMI, body mass index; eGFR, estimated glomerular filtration rate; NYHA, New York Heart Association.

Overall, the prevalence of diabetes among hospitalized patients with HF was 23.21% (95% CI: 21.39–25.13). As shown in **Figure 1**, subgroup comparisons demonstrated that the prevalence of diabetes was significantly higher in females than in males (24.94% vs 20.83%; P = 0.036) and in patients with BMI ≥24 kg/m^2^ than in those with BMI <24 kg/m^2^ (28.04% vs 21.90%; P = 0.009). No significant difference was observed between patients aged <70 years and those ≥70 years (23.44% vs 23.12%; P = 0.925). Although prevalence was higher in patients with eGFR <60 mL/min/1.73 m^2^ than in those with eGFR ≥60 mL/min/1.73 m^2^ (25.19% vs 21.55%), the difference was not statistically significant (P = 0.061). Across NYHA classes II, III, and IV, the prevalence of diabetes was 20.96%, 21.66%, and 27.11%, respectively, with a significant overall difference (P = 0.022).

### 3.3 Univariate logistic regression analysis

In the univariate logistic regression analyses (**Table 2**), sex and NYHA class showed significant associations with diabetes. Using female sex as the reference, male sex was associated with lower odds of diabetes (OR = 0.79, P = 0.032). Compared with NYHA class II, NYHA class IV was associated with higher odds of diabetes (OR = 1.40, P = 0.034). Among continuous variables, higher BMI, SBP, WBC, monocyte count, lymphocyte count, SCr, potassium, and triglycerides were associated with higher odds of diabetes, whereas higher albumin, A/G ratio, TBIL, and HDL-C were associated with lower odds of diabetes. Among medication variables, statin use was associated with higher odds of diabetes (OR = 1.69, P < 0.001). Most of the other clinical and laboratory variables were not significantly associated with diabetes in the univariate models.

**Table 2:** Univariate logistic regression.

Variable	OR	95% CI	P-value
Age, n (%)			
<70	1.00		
≥70	0.98	0.78–1.24	0.878
Sex, n (%)			
Female	1.00		
Male	0.79	0.64–0.98	0.032
Type, n (%)			
Left	1.00		
right	0.54	0.22–1.15	0.140
both	1.05	0.82–1.34	0.721
NYHA, n (%)			
II	1.00		
III	1.04	0.78–1.41	0.784
IV	1.40	1.03–1.92	0.034
BMI (kg/m²)	1.03	1.00–1.06	0.020
SBP (mmHg)	1.00	1.00–1.01	0.032
DBP (mmHg)	1.00	0.99–1.01	0.852
MAP (mmHg)	1.00	1.00–1.01	0.328
WBC (×10^9^/L)	1.08	1.05–1.11	<0.001
MONO (×10^9^/L)	1.78	1.18–2.65	0.005
RBC (×10^12^/L)	0.94	0.82–1.07	0.338
Lymphocyte (×10^9^/L)	1.28	1.09–1.51	0.002
Hemoglobin (g/L)	1.00	0.99–1.00	0.037
Platelet (×10^9^/L)	1.00	1.00–1.00	0.432
BNP (pg/mL)	1.00	1.00–1.00	0.721
hs-cTn (pg/mL)	1.02	0.96–1.07	0.493
SCr (μmol/L)	1.00	1.00–1.00	0.002
SUA (μmol/L)	1.00	1.00–1.00	0.837
eGFR (mL/min/1.73 m²)	1.00	0.99–1.00	0.143
Calcium (mmol/L)	1.15	0.64–2.04	0.642
Potassium (mmol/L)	1.31	1.14–1.51	<0.001
Albumin (g/L)	0.98	0.96–1.00	0.035
Globulin (g/L)	1.01	0.99–1.03	0.298
A/G ratio	0.65	0.46–0.91	0.012
TBIL (μmol/L)	0.99	0.98–1.00	0.005
Cholesterol (mmol/L)	0.96	0.88–1.06	0.431
LDL-C (mmol/L)	0.97	0.85–1.12	0.714
HDL-C (mmol/L)	0.56	0.41–0.75	<0.001
Triglyceride (mmol/L)	1.32	1.16–1.51	<0.001
Statins, n (%)			
NO	1.00		
YES	1.69	1.37-2.08	<0.001
ACEI/ARB, n (%)			
NO	1.00		
YES	0.92	0.74-1.14	0.467
Diuretics, n (%)			
NO	1.00		
YES	1.08	0.49-2.72	0.857
Beta-blockers, n (%)			
NO	1.00		
YES	0.98	0.80-1.22	0.887

NYHA, New York Heart Association; BMI, body mass index; SBP, systolic blood pressure; DBP, diastolic blood pressure; MAP, mean arterial pressure; WBC, white blood cell; MONO, monocyte; RBC, red blood cell; BNP, B-type natriuretic peptide; hs-cTn, high-sensitivity cardiac troponin; SCr, serum creatinine; SUA, serum uric acid; eGFR, estimated glomerular filtration rate; A/G ratio, albumin-to-globulin ratio; TBIL, total bilirubin; LDL-C, low-density lipoprotein cholesterol; HDL-C, high-density lipoprotein cholesterol; ACEI, Angiotensin-Converting Enzyme Inhibitor; ARB, Angiotensin II Receptor Blocker.

### 3.4 Multivariate logistic regression analysis

**Figure 2:**
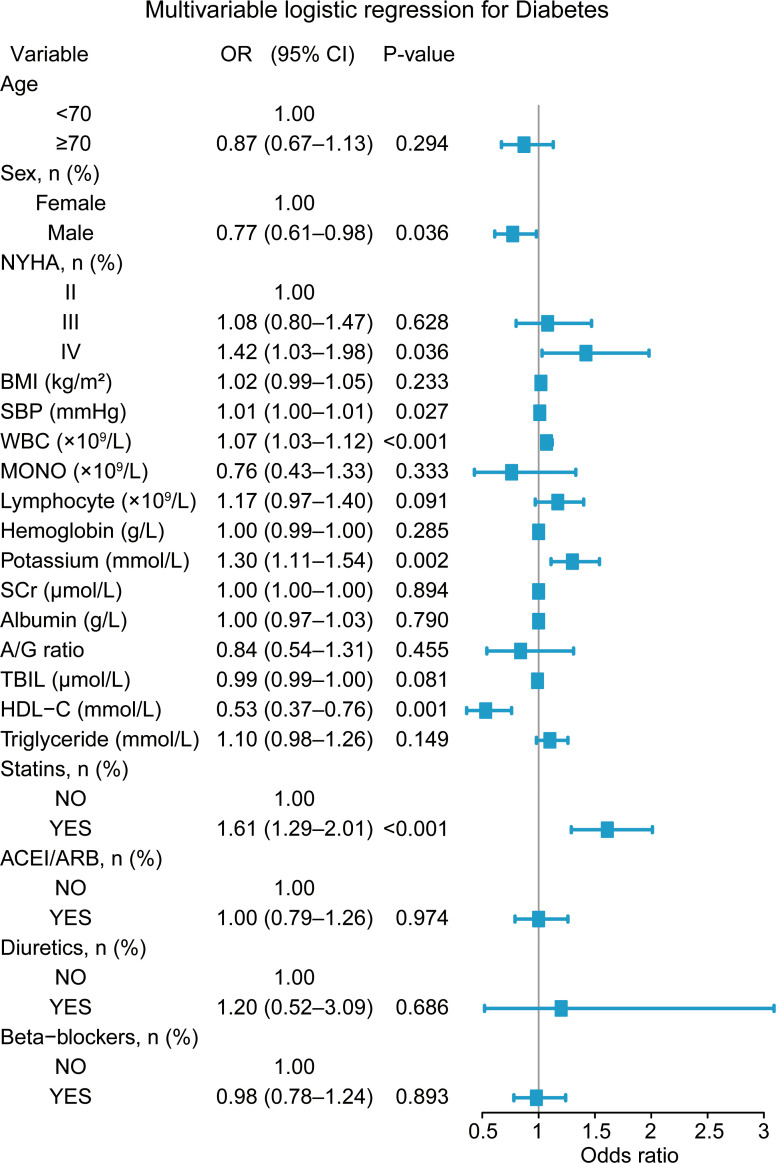
Multivariable logistic regression analysis. NYHA, New York Heart Association; BMI, body mass index; SBP, systolic blood pressure; WBC, white blood cell; Mono, monocyte; SCr, serum creatinine; A/G ratio, albumin-to-globulin ratio; TBIL, total bilirubin; HDL-C, high-density lipoprotein cholesterol, ACEI, Angiotensin-Converting Enzyme Inhibitor; ARB, Angiotensin II Receptor Blocker.

In the multivariable logistic regression model (**Figure 2**), several variables remained significantly associated with diabetes after mutual adjustment. Male sex was associated with lower odds of diabetes compared with female sex (OR = 0.77, P = 0.036). Compared with NYHA class II, NYHA class IV was positively associated with diabetes (OR = 1.42, P = 0.036), whereas class III showed no significant association. Among continuous variables, higher SBP, WBC and potassium were independently associated with increased odds of diabetes. In contrast, HDL-C was inversely associated with diabetes (OR = 0.53, P = 0.001). Statin use was positively associated with diabetes (OR = 1.61, P < 0.001), whereas ACEI/ARB use, diuretic use, and beta-blocker use were not significantly associated with diabetes. Age, BMI, hemoglobin, monocyte and lymphocyte count, SCr, albumin, A/G ratio, triglycerides and other variables were not significantly associated with diabetes. No substantial multicollinearity was observed among the included covariates, with all GVIF^(1/(2*Df)) values ranging from 1.01 to 1.37 (**Supplementary Table S1**). In addition, the model demonstrated modest discrimination (AUC = 0.659), and the ROC and calibration curves are shown in **Supplementary Figure S1**.

### 3.5 Variable importance ranking using SHAP

**Figure 3:**
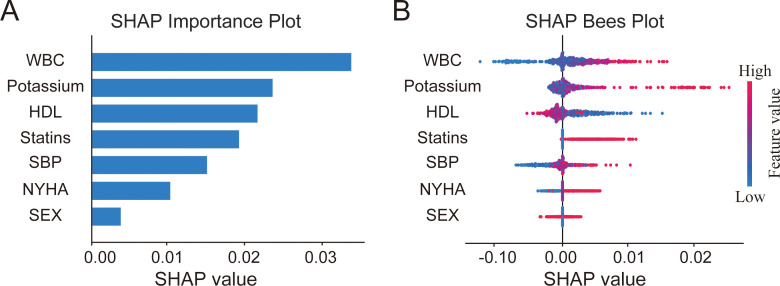
SHAP-based importance ranking of diabetes-related variables in the XGBoost model. **(A)** SHAP importance plot showing the mean absolute SHAP value for each variable in the XGBoost model. **(B)** SHAP Bees plot displaying the distribution of SHAP values for each variable across all patients.

**Figure 4:**
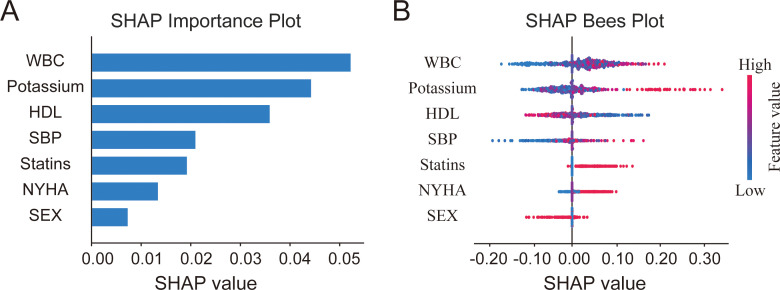
SHAP-based importance ranking of diabetes-related variables in the Random Forest model. **(A)** SHAP importance plot showing the mean absolute SHAP value for each variable in the random forest model. **(B)** SHAP Bees plot displaying the distribution of SHAP values for each variable across all patients.

To complement the multivariable logistic regression analysis, we further developed XGBoost and random forest models using the diabetes-related variables retained in the adjusted analysis and interpreted them with SHAP (**Figures 3**, **4**). In both models, WBC was ranked as the most important variable, followed by potassium and HDL-C. The SHAP distributions further showed that higher WBC and potassium levels were generally associated with higher SHAP values, whereas higher HDL-C levels showed an opposite pattern. Overall, the variable importance patterns were broadly consistent across the two algorithms. Model evaluation is shown in **Supplementary Figures S2**, **S3**. For XGBoost, the mean training AUC across five-fold cross-validation was 0.840, and the test-set AUC was 0.622. For the random forest model, the mean training AUC across five-fold cross-validation was 0.872, and the test-set AUC was 0.656. In addition, the calibration plots of both models showed acceptable but not perfect agreement with the ideal line in the test set.

### 3.6 Nonlinear associations with diabetes

**Figure 5:**
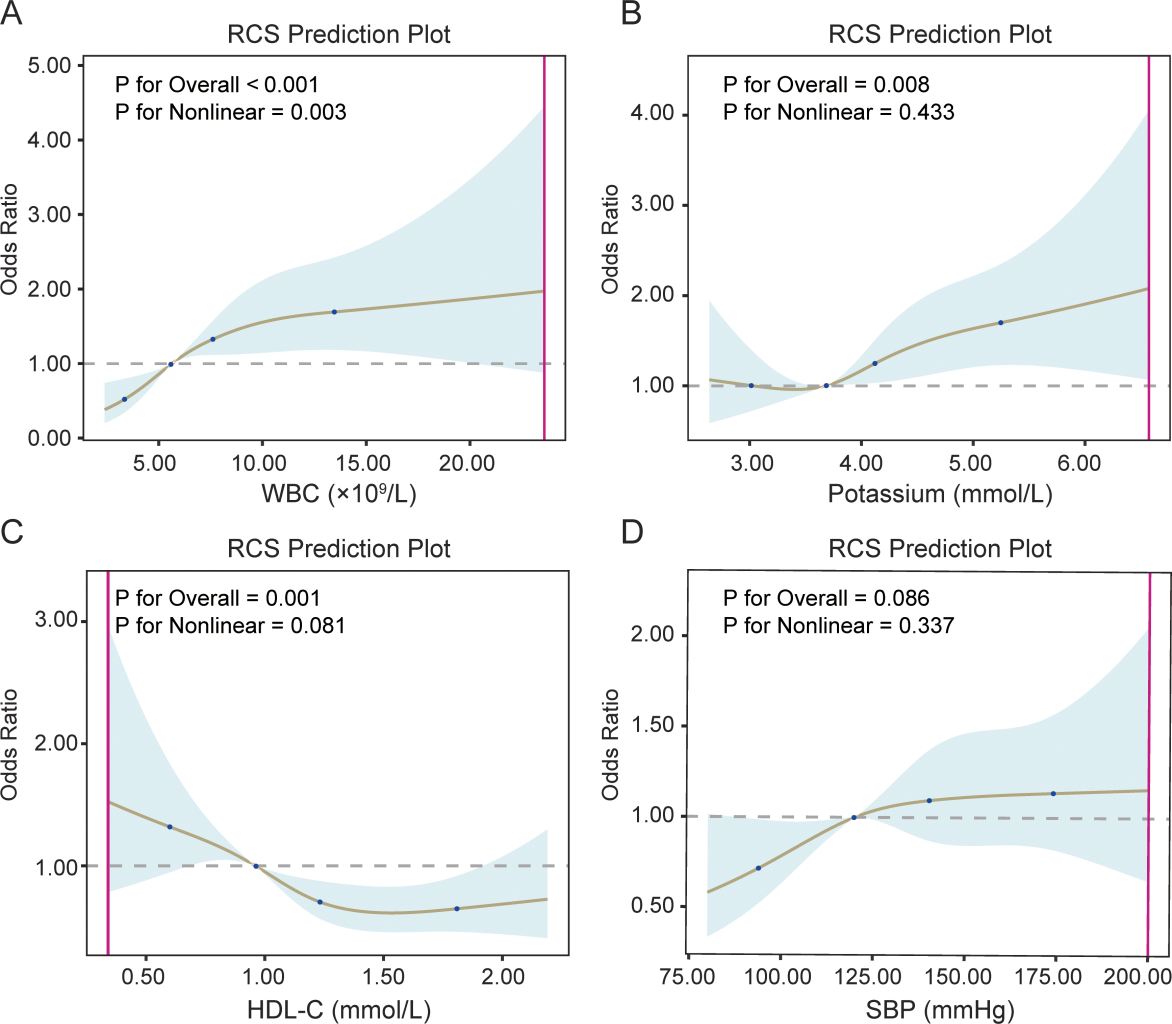
Nonlinear associations of continuous variables with diabetes. **(A)** Restricted cubic spline curve for the association between WBC and Diabetes. **(B)** Restricted cubic spline curve for the association between potassium and Diabetes. **(C)** Restricted cubic spline curve for the association between HDL-C and Diabetes. **(D)** Restricted cubic spline curve for the association between SBP and Diabetes.

In restricted cubic spline analyses, WBC, HDL-C, and potassium showed significant overall associations with the odds of diabetes, whereas SBP did not (**Figure 5**). WBC displayed a clear nonlinear relationship with diabetes (P for overall <0.001, P for nonlinearity = 0.003), with the odds of diabetes increasing steeply as WBC rose from lower to intermediate levels and then plateauing at higher counts. For HDL-C and potassium, the overall associations with diabetes were statistically significant, but there was no clear evidence of marked nonlinearity. In contrast, SBP did not show a statistically significant overall association or nonlinear association with diabetes.

### 3.7 Sex and age stratified analyses

In the sex-stratified analysis (**Table 3**), among females, NYHA class IV remained significantly associated with higher odds of diabetes (OR = 1.56, 95% CI: 1.04–2.38), and higher WBC and potassium levels were positively related to diabetes, whereas HDL-C was inversely associated (OR = 0.44, 95% CI: 0.27–0.71). Statin use was also significantly associated with diabetes among females (OR = 1.48, 95% CI: 1.11–1.97). In males, statin use was significantly associated with diabetes (OR = 1.90, 95% CI: 1.33–2.73), whereas the other variables showed no clear associations. Formal interaction analyses further showed no statistically significant interactions between sex and SBP, NYHA class, or HDL-C (all P for interaction > 0.05). In the age-stratified analysis (**Table 4**), higher SBP and lower albumin were associated with diabetes in patients <70 years. Statin use was also associated with diabetes in this age group (OR = 1.62, 95% CI: 1.03–2.53). In contrast, among patients ≥70 years, WBC, potassium, and triglycerides were positively associated with diabetes (WBC: OR = 1.09, 95% CI: 1.03–1.14; potassium: OR = 1.38, 95% CI: 1.14–1.67; triglycerides: OR = 1.27, 95% CI: 1.04–1.59), while HDL-C remained negatively associated with diabetes (OR = 0.44, 95% CI: 0.28–0.67). Statin use was also associated with diabetes among patients aged ≥70 years (OR = 1.57, 95% CI: 1.21–2.04).

**Table 3:** Sex stratified correlates of diabetes in HF patients.

Variable	Female			Male		
	OR	95% CI	P-value	OR	95% CI	P-value
Age, n (%)						
<70	1.00			1.00		
≥70	0.93	0.66-1.33	0.689	0.85	0.56-1.31	0.467
NYHA, n (%)						
II	1.00			1.00		
III	1.11	0.76-1.63	0.604	1.02	0.62-1.73	0.936
IV	1.56	1.04-2.38	0.035	1.20	0.70-2.09	0.518
BMI (kg/m²)	1.01	0.97-1.04	0.735	1.04	0.99-1.09	0.093
SBP (mmHg)	1.01	1.00-1.01	0.049	1.00	1.00-1.01	0.380
WBC (×10^9^/L)	1.08	1.03-1.14	0.002	1.06	0.99-1.14	0.091
MONO (×10^9^/L)	0.76	0.36-1.57	0.463	0.70	0.27-1.80	0.461
Lymphocyte (×10^9^/L)	1.15	0.90-1.46	0.264	1.24	0.93-1.66	0.146
Hemoglobin (g/L)	1.00	0.99-1.00	0.308	1.00	0.99-1.01	0.534
Potassium (mmol/L)	1.36	1.11-1.67	0.003	1.17	0.88-1.55	0.274
SCr (μmol/L)	1.00	1.00-1.00	0.653	1.00	1.00-1.00	0.395
Albumin (g/L)	0.99	0.96-1.03	0.748	1.01	0.96-1.06	0.675
A/G ratio	0.66	0.36-1.19	0.171	1.17	0.58-2.35	0.663
TBIL (μmol/L)	1.00	0.99-1.00	0.410	0.99	0.97-1.00	0.060
HDL-C (mmol/L)	0.44	0.27-0.71	0.001	0.79	0.44-1.39	0.430
Triglyceride (mmol/L)	1.08	0.95-1.26	0.291	1.17	0.84-1.63	0.344
Statins, n (%)						
NO	1.00			1.00		
YES	1.48	1.11-1.97	0.008	1.90	1.33-2.73	<0.001
ACEI/ARB, n (%)						
NO	1.00			1.00		
YES	1.00	0.73-1.36	0.999	1.00	0.69-1.45	0.997
Diuretics, n (%)						
NO	1.00			1.00		
YES	1.27	0.44-4.60	0.688	1.21	0.34-5.70	0.789
Beta-blockers, n (%)						
NO	1.00			1.00		
YES	0.93	0.68-1.26	0.641	1.13	0.78-1.63	0.506

NYHA, New York Heart Association; BMI, body mass index; SBP, systolic blood pressure; WBC, white blood cell; MONO, monocyte; SCr, serum creatinine; A/G ratio, albumin-to-globulin ratio; TBIL, total bilirubin; HDL-C, high-density lipoprotein cholesterol; ACEI, Angiotensin-Converting Enzyme Inhibitor; ARB, Angiotensin II Receptor Blocker.

**Table 4:** Age-stratified correlates of diabetes in HF patients.

Variable	<70			≥70		
	OR	95% CI	P-value	OR	95% CI	P-value
Sex, n (%)						
Female	1.00			1.00		
Male	0.79	0.49-1.28	0.339	0.80	0.60-1.07	0.130
NYHA, n (%)						
II	1.00			1.00		
III	1.34	0.73-2.54	0.356	1.03	0.72-1.48	0.890
IV	1.99	1.04-3.97	0.043	1.31	0.89-1.93	0.176
BMI (kg/m²)	1.03	0.98-1.09	0.214	1.01	0.97-1.04	0.633
SBP (mmHg)	1.01	1.00-1.02	0.004	1.00	1.00-1.01	0.510
WBC (×10^9^/L)	1.03	0.95-1.11	0.461	1.09	1.03-1.14	0.001
MONO (×10^9^/L)	1.13	0.40-3.23	0.814	0.67	0.33-1.35	0.266
Lymphocyte (×10^9^/L)	1.28	0.91-1.82	0.157	1.13	0.90-1.41	0.287
Hemoglobin (g/L)	1.00	0.99-1.01	0.556	1.00	0.99-1.00	0.259
Potassium (mmol/L)	1.02	0.74-1.41	0.896	1.38	1.14-1.67	0.001
SCr (μmol/L)	1.00	1.00-1.01	0.284	1.00	1.00-1.00	0.692
Albumin (g/L)	0.94	0.89-0.99	0.026	1.02	0.98-1.06	0.283
A/G ratio	0.91	0.40-2.07	0.827	0.80	0.46-1.37	0.418
TBIL (μmol/L)	1.00	0.99-1.01	0.737	0.99	0.98-1.00	0.115
HDL-C (mmol/L)	0.98	0.50-1.86	0.953	0.44	0.28-0.67	<0.001
Triglyceride (mmol/L)	1.06	0.90-1.23	0.470	1.27	1.04-1.59	0.031
Statins, n (%)						
NO	1.00			1.00		
YES	1.62	1.03-2.53	0.037	1.57	1.21-2.04	0.001
ACEI/ARB, n (%)						
NO	1.00			1.00		
YES	0.95	0.60-1.48	0.809	0.99	0.75-1.31	0.958
Diuretics, n (%)						
NO	1.00			1.00		
YES	0.73	0.12-5.88	0.743	1.48	0.57-4.59	0.451
Beta-blockers, n (%)						
NO	1.00			1.00		
YES	1.20	0.77-1.89	0.420	0.90	0.68-1.19	0.468

NYHA, New York Heart Association; BMI, body mass index; SBP, systolic blood pressure; WBC, white blood cell; MONO, monocyte; SCr, serum creatinine; A/G ratio, albumin-to-globulin ratio; TBIL, total bilirubin; HDL-C, high-density lipoprotein cholesterol; ACEI, Angiotensin-Converting Enzyme Inhibitor; ARB, Angiotensin II Receptor Blocker.

## 4 Discussion

We found that the prevalence of diabetes was 23.21% among HF inpatients. In the adjusted analysis, female sex, NYHA class IV, higher SBP, higher WBC count, higher serum potassium, and statin use were associated with greater odds of diabetes, whereas higher HDL-C was independently and inversely associated with diabetes. Complementary machine learning analyses identified WBC, potassium, and HDL-C as among the most important variables in both the XGBoost and random forest models. Restricted cubic spline analyses further suggested a clear nonlinear association between WBC and diabetes. In addition, sex- and age-stratified models indicated that these relationships were not uniform across subgroups: the association between diabetes and advanced NYHA class appeared more evident in females, the link with higher potassium was stronger in older patients, and the inverse association between HDL-C and diabetes was particularly robust in these subgroups.

The prevalence of diabetes in our cohort of hospitalized patients with HF was 23.21%, broadly in line with contemporary registry data and prior literature. In the nationwide Swedish Heart Failure Registry, 22.00% of patients had diabetes, very similar to our estimate (15). Likewise, a study from Colombia of hospitalized HF patients reported a diabetes prevalence of 24.7%, again closely matching our findings (16). Large reviews of the HF literature also suggest that roughly 25–40% of HF patients have concomitant diabetes, placing our data at the lower end of this expected range (17). In contrast, some cohorts have reported substantially higher prevalences: for example, the Jordanian Heart Failure Registry found that nearly 70% of HF patients had diabetes (18), and HF populations enriched for mildly reduced or preserved ejection fraction often show diabetes rates around 37-43% (19, 20). Randomized HF trials also tend to include a higher proportion of patients with diabetes. For instance, diabetes was present in about 62% of participants in the CHARM program and in approximately 49.8% of those enrolled in the PARAGON-HF trial (21). Moreover, a recent analysis showed that the proportion of HF hospitalizations occurring in patients with diabetes has increased over the past two decades, underscoring the growing overlap between the two conditions (22). Taken together, these data indicate that the diabetes burden observed in our Chinese HF cohort is comparable to that seen in many other real-world HF populations, while somewhat lower than in highly comorbid or trial selected cohorts.

We found that the prevalence of diabetes was lower in males than in females among patients with heart failure. In the multivariable model, male sex was associated with lower odds of diabetes, suggesting a higher diabetes burden among female patients in this hospitalized HF cohort. A large meta-analysis showed that diabetes was more strongly associated with heart failure in women than in men (23). In another heart failure cohort, women were more likely to have diabetes despite a lower body mass index and had a greater burden of chronic kidney disease than men (24). A further study also reported a higher prevalence of diabetes in women than in men with heart failure, with rates of 60% versus 55% (25). Previous studies suggest that although diabetes is more prevalent in males than in females among young and middle-aged adults, females may have a higher overall prevalence of diabetes after 70 years of age (26). As the patients included in our study were predominantly older adults, this age-related pattern may be relevant to the observed sex difference. Although women generally have more favorable insulin sensitivity earlier in life, this pattern may become less apparent with aging, menopause, and worsening glucose tolerance (27). Women may also show a greater burden of adverse metabolic changes before overt diabetes, including less favorable adiposity, blood pressure, glucose, and lipid profiles (28). In addition, older women are more likely to present with postprandial hyperglycemia, and menopause-related increases in visceral adiposity may also be relevant (29). Behavioral and treatment-related gender differences may likewise be associated with susceptibility to diabetes and its progression.

In the present study, WBC count was independently and positively associated with diabetes among patients with HF (OR = 1.07, 95% CI: 1.03–1.12). Restricted cubic spline analyses suggested a nonlinear pattern in this association, and WBC also ranked as the top contributor in both machine learning models. Previous studies have similarly reported positive associations between higher WBC levels and diabetes-related outcomes. For example, Twig and colleagues reported that every 1,000 cells/mm^3^ increment in WBC was associated with a 7.6% higher incidence of diabetes, even though values remained within the conventional normal range, and that WBC remained associated with diabetes after adjustment for BMI and other metabolic risk factors (30). The Yuport Medical Checkup Center Study also reported that higher baseline WBC was associated with incident diabetes in Japanese adults, and that the combination of elevated WBC and C-reactive protein was related to a higher risk estimate (31). Furthermore, a large Chinese cohort showed that higher total WBC, neutrophil and lymphocyte counts were all associated with both newly diagnosed and future type 2 diabetes and correlated positively with HbA1c, reinforcing the link between leukocytosis, insulin resistance and chronic hyperglycemia (32). Taken together, these findings suggest that WBC may reflect an inflammatory state associated with abnormal glucose metabolism in patients with HF. However, given the cross-sectional design of the present study, the temporal direction of this association cannot be determined.

Higher serum potassium and statin use were both independently associated with diabetes. This finding is broadly consistent with previous literature. Collins et al. showed that potassium abnormalities were more common in patients with HF, CKD, or diabetes, reflecting the close overlap among these conditions (33). In addition, prior observational studies have reported that statin initiation was associated with a higher likelihood of diabetes progression (34). However, these associations should be interpreted cautiously in the context of our cross-sectional design. Because the kidney plays a central role in potassium homeostasis, impaired renal potassium excretion in the setting of kidney dysfunction may contribute to higher serum potassium levels (35). In addition, renin–angiotensin–aldosterone system-inhibiting therapies commonly used in HF may increase serum potassium by reducing aldosterone-mediated potassium excretion, whereas the effects of diuretics may vary by class, with loop and thiazide diuretics tending to promote potassium loss and potassium-sparing agents having the opposite effect (36, 37). Patients with heart failure may also experience acid-base disturbances, which can further alter transcellular potassium distribution and thereby complicate the interpretation of a single serum potassium measurement (38). Therefore, the association between potassium and diabetes in our study may be better understood as reflecting overlapping comorbidity burden, treatment exposure, and acute physiological disturbances.

In our cohort, higher SBP was associated with greater odds of diabetes. This observation is broadly consistent with previous studies reporting a positive relationship between SBP and diabetes-related outcomes (39). In addition, another study reported positive associations of both SBP and DBP with incident type 2 diabetes (40). Several biological pathways may partly explain this association. Previous studies have suggested that elevated blood pressure may be accompanied by low-grade inflammation, oxidative stress, endothelial dysfunction, and impaired microvascular perfusion, all of which may be relevant to glucose metabolism (41, 42). In addition, higher blood pressure often coexists with obesity, neurohormonal activation, and other adverse cardiometabolic features, which may also contribute to the observed association (43, 44). Overall, our findings are consistent with previous studies showing a positive association between SBP and diabetes. However, residual confounding related to obesity, medication use, and underlying cardiometabolic status may also have influenced this association.

In the adjusted model, higher HDL-C was independently associated with lower odds of diabetes, with an OR of 0.53 in the adjusted model. HDL-C was also ranked among the top three variables in the SHAP analysis, and the restricted cubic spline curves suggested a largely monotonic inverse association between HDL-C and diabetes. These findings are generally consistent with previous observational studies. For example, a recent analysis of 9,420 adults from NHANES reported lower odds of diabetes among individuals in the highest HDL-C quartile than among those in the lowest quartile, and the spline analysis also showed an overall decreasing pattern across increasing HDL-C levels (45). Prospective cohort data from Asia provided further support. In a large Japanese cohort of more than 15,000 adults, lower HDL-C was associated with a higher incidence of diabetes, and a nonlinear relationship was also described (46). A meta-analysis of 15 studies reported that patients with type 2 diabetes had markedly lower HDL-C than controls, reinforcing low HDL-C as a typical feature of diabetic dyslipidemia (47). In addition, a prospective study in individuals with prediabetes reported that a higher triglyceride-to-HDL-C ratio was associated with a higher incidence of diabetes (48).

Previous experimental and clinical studies have suggested that HDL-C may be involved in several mechanisms relevant to glucose homeostasis. HDL-C particles and their major apolipoprotein, apolipoprotein A-I, have been reported to support pancreatic β-cell function and insulin secretion in experimental studies (49). In peripheral tissues, HDL-C and ApoA-I have also been linked to increased glucose uptake and improved metabolic regulation (50). HDL-C has additionally been associated with anti-inflammatory and antioxidant properties that may be relevant to insulin sensitivity and glucose regulation (51). At the same time, HDL-C function may be impaired in diabetes, and so-called dysfunctional HDL-C has been described in the setting of glycation, oxidation, and inflammatory modification (52). These mechanisms may provide biological plausibility for the observed association; however, in the present cross-sectional study, they should be regarded as possible explanations rather than direct evidence for a directional relationship between HDL-C and diabetes.

The present study combined clinical characteristics, hemodynamic indices and routinely measured blood biomarkers to explore factors associated with diabetes in HF patients. Moreover, by combining multivariable logistic regression with machine learning methods and restricted cubic spline analyses, we obtained a more detailed picture of these associations in a hospitalized HF cohort, which has been rarely reported in Chinese populations. However, several limitations should be noted. First, as a cross-sectional study based on prevalent diabetes, our findings can only be interpreted as associations rather than causal relationships between diabetes and the identified variables. Second, diabetes status in this study was identified from prior medical history information documented in the admission notes at hospital admission rather than from a standardized adjudication protocol. The original records did not further specify the diagnostic basis for pre-existing diabetes, nor did they provide detailed information on glucose-lowering medication use or ICD coding. In addition, systematic HbA1c or oral glucose tolerance testing data were not available in the source records. Therefore, previously undiagnosed diabetes may have been missed, and transient stress hyperglycemia during acute hospitalization may not have been fully distinguishable from established diabetes. As a result, some degree of misclassification is possible, which may have affected both the estimated prevalence of diabetes and the observed associations with clinical variables. Third, although available medication variables, including statins, ACEI/ARB, diuretics, and beta-blockers, were included in the analysis, detailed other treatment details were unavailable. Therefore, residual confounding related to medication use cannot be excluded. In addition, because LVEF was missing in more than 60% of the study sample, we were unable to evaluate differences in diabetes across HF phenotypes. Finally, the data were derived from a single tertiary hospital and included only hospitalized HF patients, which may limit generalizability and leave room for residual confounding.

## 5 Conclusion

Diabetes was common among hospitalized patients with HF in this cohort. Several clinical characteristics, including female sex, NYHA class IV, higher SBP, higher WBC count, higher serum potassium, statin use, and lower HDL-C, were associated with diabetes in the adjusted analyses. These findings may help improve understanding of the clinical profile of HF patients with coexisting diabetes. However, given the cross-sectional design, possible misclassification, residual confounding, and the single-center inpatient setting, these results should be interpreted cautiously. Further studies are needed to confirm these associations and clarify their clinical relevance.

## Data Availability

Publicly available datasets were analyzed in this study. This data can be found here: https://physionet.org/content/heart-failure-zigong/1.3/.

## Supplementary material

Supplementary Table S1 Multicollinearity diagnostics for variables included in the multivariable logistic regression model. NYHA, New York Heart Association; BMI, body mass index; SBP, systolic blood pressure; WBC, white blood cell; MONO, monocyte; SCr, serum creatinine; A/G ratio, albumin-to-globulin ratio; TBIL, total bilirubin; HDL-C, high-density lipoprotein cholesterol; ACEI, Angiotensin-Converting Enzyme Inhibitor; ARB, Angiotensin II Receptor Blocker.

Supplementary Figure S1 Receiver operating characteristic curve and calibration curve of the multivariable logistic regression model. **(A)** ROC curve. **(B)** Calibration curve.

Supplementary Figure S2 Performance evaluation of the XGBoost model. **(A)** ROC curves of the XGBoost model in the training set based on five-fold cross-validation. **(B)** ROC curve of the XGBoost model in the test set. **(C)** Calibration plot of the XGBoost model in the test set.

Supplementary Figure S3 Performance evaluation of the Random Forest model. **(A)** ROC curves of the Random Forest model in the training set based on five-fold cross-validation. **(B)** ROC curve of the Random Forest model in the test set. **(C)** Calibration plot of the Random Forest model in the test set.
