# Supplementary material for: Prevalence and clinical correlates of diabetes in hospitalized heart failure patients: a retrospective study
Source: Front Endocrinol (Lausanne). 2026 Apr 23;17:1806046. doi: 10.3389/fendo.2026.1806046 (PMC13149073; doi:10.3389/fendo.2026.1806046)
Supplement: Supplementary Table S1 — Multicollinearity diagnostics for variables included in the multivariable logistic regression model. NYHA, New York Heart Association; BMI, body mass index; SBP, systolic blood pressure; WBC, white blood cell; MONO, monocyte; SCr, serum creatinine; A/G ratio, albumin-to-globulin ratio; TBIL, total bilirubin; HDL-C, high-density lipoprotein cholesterol; ACEI, Angiotensin-Converting Enzyme Inhibitor; ARB, Angiotensin II Receptor Blocker. [file Table1.docx]

| Table S1. Multicollinearity diagnostics for variables included in the multivariable logistic regression model | |
| --- | --- |
| Variable | GVIF^(1/(2*Df)) |
| Age | 1.08 |
| Sex | 1.09 |
| NYHA | 1.02 |
| BMI | 1.03 |
| SBP | 1.09 |
| WBC | 1.37 |
| MONO | 1.35 |
| Lymphocyte | 1.09 |
| Hemoglobin | 1.25 |
| Potassium | 1.10 |
| SCr | 1.18 |
| Albumin | 1.37 |
| A/G ratio | 1.29 |
| TBIL | 1.12 |
| HDL-C | 1.14 |
| Triglyceride | 1.10 |
| Statins | 1.04 |
| ACEI/ARB | 1.05 |
| Diuretics | 1.01 |
| Beta-blockers | 1.06 |

Abbreviations: NYHA, New York Heart Association; BMI, body mass index; SBP, systolic blood pressure; WBC, white blood cell; MONO, monocyte; SCr, serum creatinine; A/G ratio, albumin-to-globulin ratio; TBIL, total bilirubin; HDL-C, high-density lipoprotein cholesterol; ACEI, Angiotensin-Converting Enzyme Inhibitor; ARB, Angiotensin II Receptor Blocker.
