# Supplementary figures and images for: Prevalence and clinical correlates of diabetes in hospitalized heart failure patients: a retrospective study
Source: Front Endocrinol (Lausanne). 2026 Apr 23;17:1806046. doi: 10.3389/fendo.2026.1806046 (PMC13149073; doi:10.3389/fendo.2026.1806046)

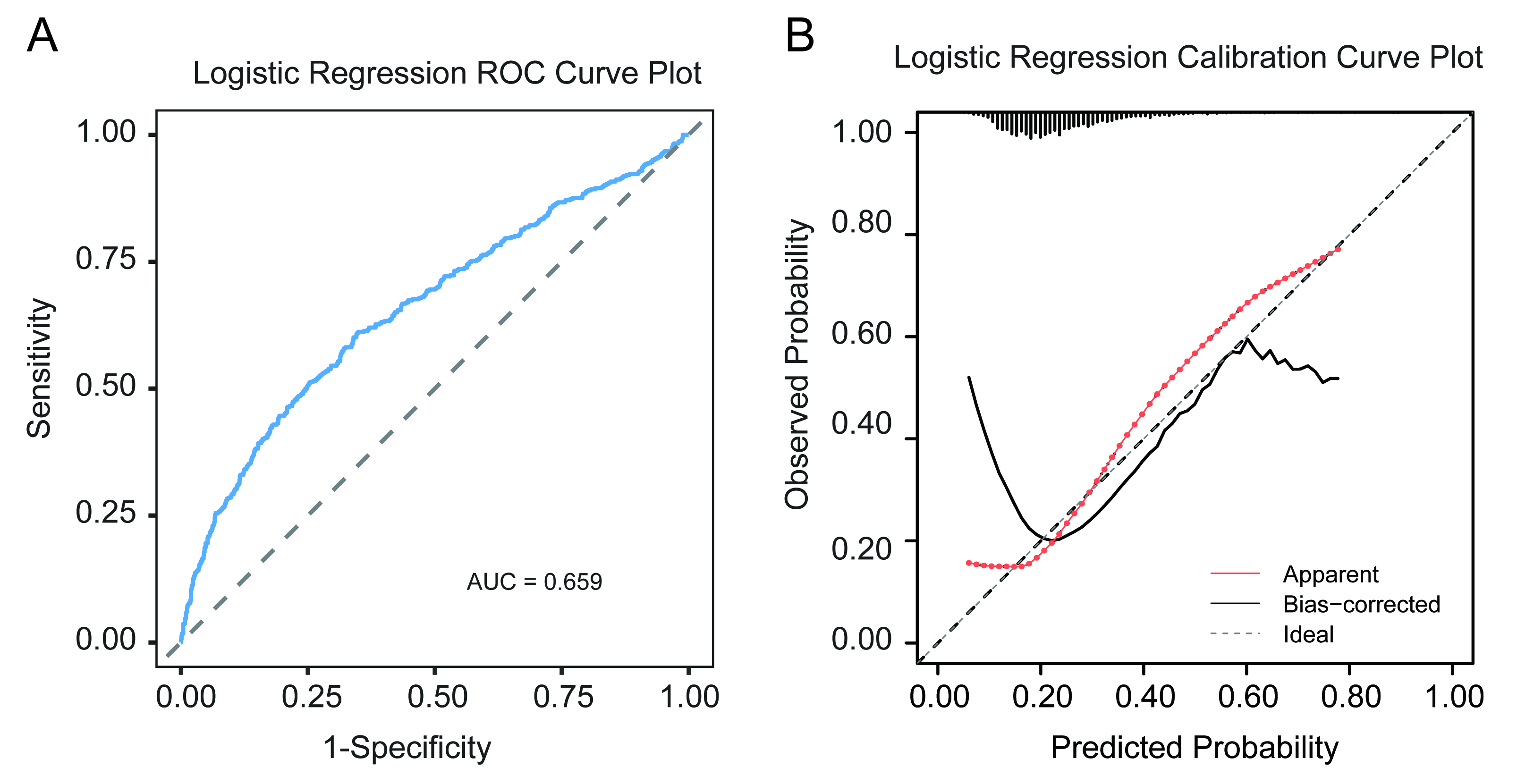

Supplement: Supplementary Figure S1 — Receiver operating characteristic curve and calibration curve of the multivariable logistic regression model. (A) ROC curve. (B) Calibration curve. [file Image1.tif]

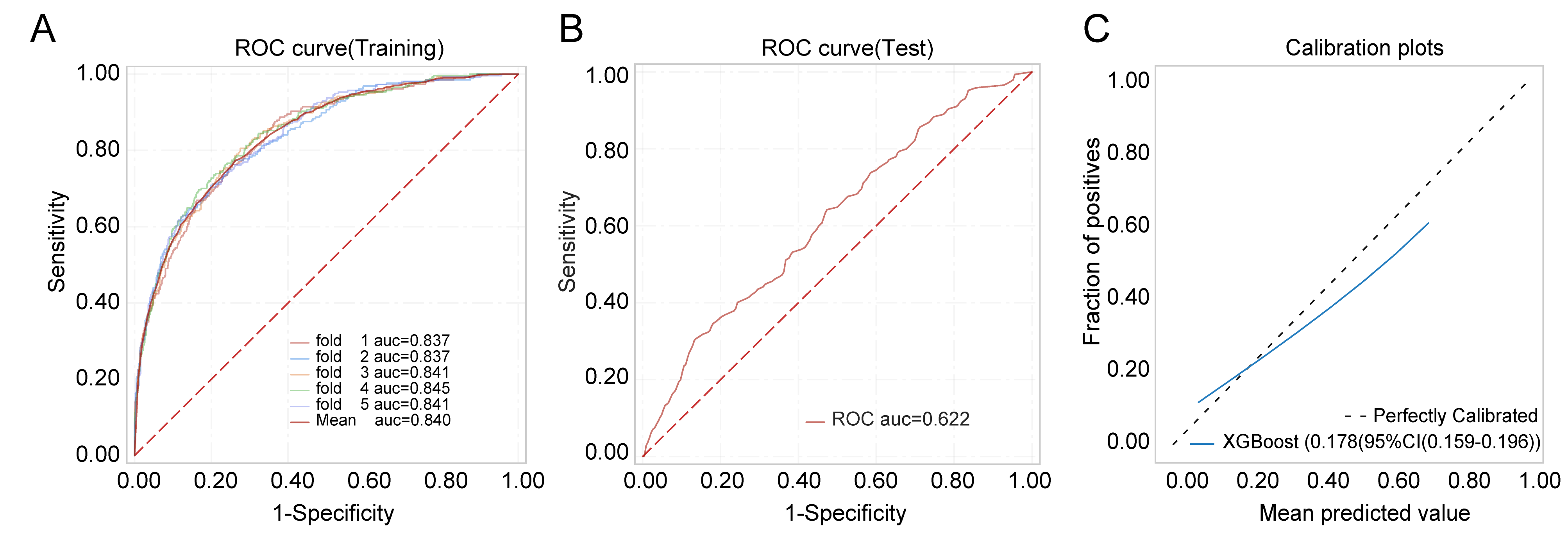

Supplement: Supplementary Figure S2 — Performance evaluation of the XGBoost model. (A) ROC curves of the XGBoost model in the training set based on five-fold cross-validation. (B) ROC curve of the XGBoost model in the test set. (C) Calibration plot of the XGBoost model in the test set. [file Image2.tif]

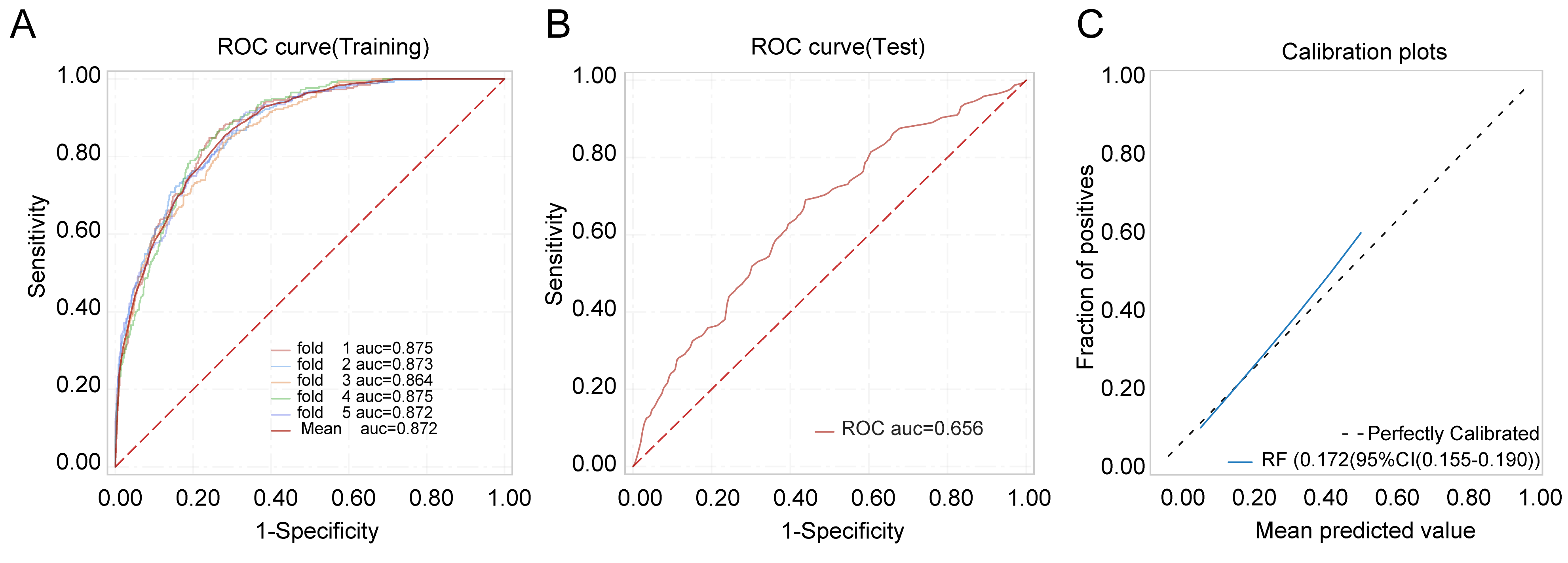

Supplement: Supplementary Figure S3 — Performance evaluation of the Random Forest model. (A) ROC curves of the Random Forest model in the training set based on five-fold cross-validation. (B) ROC curve of the Random Forest model in the test set. (C) Calibration plot of the Random Forest model in the test set. [file Image3.tif]
